# Effects of Gamma Irradiation and Heat Treatment on the ATR-FTIR Response and Shore A Hardness of Silicone Rubber-Based Composites

**DOI:** 10.3390/nano16201286

**Published:** 2026-10-10

**Authors:** Guang Hu, Fu Ji, Yihong Yan, Xu Wang, Chao Wang, Lingzhi Tang, Junqi Wang, Weiqiang Sun

**Affiliations:** 1 School of Nuclear Science and Technology, Xi’an Jiaotong University, Xi’an 710049, China; guanghu@xjtu.edu.cn (G.H.); jifu0822@stu.xjtu.edu.cn (F.J.); hngcwangx@stu.xjtu.edu.cn (X.W.); wang_chao@stu.xjtu.edu.cn (C.W.); lingzhi2021@stu.xjtu.edu.cn (L.T.); 2 School of Human Settlements and Civil Engineering, Xi’an Jiaotong University, Xi’an 710049, China; yihong@xjtu.edu.cn

**Keywords:** silicone-based composites, gamma irradiation, post-irradiation annealing, ATR-FTIR, EPR, Shore A hardness

## Abstract

Silicone rubber (SR) composites are candidate radiation-shielding materials, but their irradiation and annealing responses remain incompletely understood. Three predefined formulations containing boron carbide (B_4_C), lead (Pb)/B_4_C, or Pb were exposed to 100 kGy gamma irradiation in air and then annealed under nitrogen at 60–120 °C for 30–240 min. Attenuated total reflectance Fourier-transform infrared (ATR-FTIR) spectra retained the same characteristic band positions after irradiation, although their normalized band areas changed. Shore A hardness increased after irradiation in all formulations and remained above the corresponding pre-irradiation values after annealing. Electron paramagnetic resonance (EPR) spectra of the SR-B_4_C formulation varied qualitatively with the annealing conditions, but absolute radical quantification and assignment of a unique reaction pathway were not possible. Because filler identity, filler loading, and silicone content varied simultaneously, differences among the formulations cannot be independently attributed to Pb, B_4_C, or filler–matrix interactions. Based on one independently prepared parent specimen per formulation, these results describe irradiation and annealing responses in composites with micrometer-sized fillers. They may inform future comparisons with silicone-based shielding nanocomposites under matched compositions and treatment conditions.

## 1. Introduction

Flexible polymer composites have been proposed for potential radiation-shielding applications in nuclear facilities, radiotherapy systems, linear accelerators, and aerospace equipment [1,2]. In such applications, attenuation, bulk mechanical behavior, and durability would require independent qualification. In marine nuclear installations, safety-enclosure and containment boundaries include piping and electrical penetrations and personnel-access openings [3,4]. Applicable rules require these features to maintain specified boundary integrity and leak-tightness under defined operating and accident conditions [3,4]. These locations provide a potential application context for conformable materials, but the present study does not assess compliance with those requirements. Silicone rubber (SR) has thermal resistance, electrical insulation, chemical stability, and flexibility that support its use in cable insulation and seals [5,6,7]. Gamma irradiation induces chain scission, crosslinking, oxidation, and side-group reactions in SR [8,9]. The relative dominance of these reactions is dose-dependent: at 50–100 kGy, scission and crosslinking compete, whereas at 250–400 kGy, crosslinking and elastomer formation become significant [10,11]. These chemical changes ultimately lead to hardening, embrittlement, and altered electrical performance. In filled SR systems, filler composition, loading, dispersion, and the filler–matrix interface can influence local structure and measured response [12,13]. Establishing these relationships requires measurements that distinguish chemical changes from bulk mechanical and shielding performance.

High-atomic-number fillers—such as PbO, Bi_2_O_3_, and WO_3_—enhance density and photon attenuation, whereas boron-containing fillers like B_4_C and boron nitride (BN) improve neutron shielding [14,15]. For instance, surface-modified BN and silica-coated BN nanosheets have been reported to improve interfacial bonding and thermal stability in SR composites [13,15]. However, the effectiveness of each filler depends on multiple factors, including its composition, particle size, loading level, dispersion quality, and interfacial adhesion to the SR matrix. High filler loading restricts chain mobility and may introduce interfacial defects or stress concentration [12,13]. Moreover, irradiation can induce structural disorder, defect accumulation, and dielectric changes in B_4_C [16,17]. Such changes may compromise interface stability and, consequently, the macroscopic performance of the composite. Previous studies have shown that heat treatment, either alone or in combination with radiation, can alter crosslinking density, electrical insulation, and aging behavior in SR [5,8]. Heat treatment may also remove some irradiation-induced defects in B_4_C [18]. However, the combined effects of post-irradiation heating time and temperature on the properties of filled SR composites have not been systematically investigated. This gap is especially important for highly filled Pb/B_4_C/SR composites intended for radiation-shielding applications. Although high filler loadings necessarily reduce some flexibility, these composites retain sufficient mechanical compliance for practical handling and installation, while providing the attenuation efficiency required for shielding applications [19]. To cover representative shielding functions—neutron, mixed neutron/photon, and photon—three formulations were designed with different combinations of Pb and B_4_C fillers. The 100 kGy dose and 60–120 °C annealing range used in this work are relevant to cumulative doses and operating temperatures encountered by shielding materials in marine nuclear environments.

In this work, we examined these three silicone-based composites: SR-B_4_C, SR-Pb-B_4_C, and SR-Pb. Samples were exposed to 100 kGy gamma irradiation and subsequently annealed under nitrogen at 60, 90, or 120 °C for 30–240 min. ATR-FTIR was used to monitor relative changes in selected Si–O–Si, Si–CH_3_, Si–(CH_3_)_2_, and C–H band areas. Shore A hardness was used as a descriptive measure of resistance to local indentation. In the context of movable shielding joints, hardness provides a practical indicator of whether the material retains sufficient compliance to accommodate mechanical movement after irradiation. EPR provided qualitative information on paramagnetic signals in SR-B_4_C. The three formulations were examined as complete systems, without attempting to separate the contributions of individual fillers.

## 2. Materials and Methods

### 2.1. Materials

Lead (Pb) powder (Top Metal Materials Co., Ltd., Xingtai, China, batch No. 20210731, 300 mesh; nominal sieve opening ≈48 μm as specified by the supplier; 99.99% purity) and boron carbide (B_4_C) powder (Top Metal Materials Co., Ltd., batch No. 20210430, 300 mesh; nominal sieve opening ≈48 μm as specified by the supplier; 99.9% purity) were used as fillers. The supplier did not report a mean particle diameter or particle-size distribution, and these quantities were not independently measured. The Pb and B_4_C powders were used as received without further purification.

The silicone base materials (vinyl-terminated polydimethylsiloxane and methyl-hydrogen silicone oil) were supplied by Shenzhen Guoyuan Technology Co., Ltd. (Shenzhen, China; batch No. 20210712). Detailed specifications—including molecular weight, viscosity, reactive group contents, and the Si–H to vinyl molar ratio—were not disclosed by the supplier for proprietary reasons. The supplier also provided no information on whether the as-received silicone base contains additional components such as reinforcing silica, inhibitors, or stabilizers. The formulations prepared in this work were composed only of the silicone base, Karstedt catalyst, and the fillers listed in Table 1 as added components. The silicone base materials and Karstedt catalyst were also used as received without further purification or pretreatment.

### 2.2. Composite Preparation

The laboratory temperature was maintained at 25 °C. Methylhydrogen silicone oil and vinyl-terminated polydimethylsiloxane were mixed at a mass ratio of 3:7. Subsequently, Karstedt catalyst was added at a concentration of 0.2 wt% relative to the total silicone base to initiate the hydrosilylation curing reaction. The SR, Pb powder, and B_4_C powder were weighed according to the compositions listed in Table 1. Each filler was added in multiple portions, and the mixture was mechanically stirred at 600 rpm for 20 min. The mixture was subsequently degassed under a vacuum of −0.095 MPa (gauge pressure) for 8 min. The degassed mixture was cast into molds and cured at 25 °C under ambient pressure for 24 h. After demolding, the samples were conditioned at 25 °C for 240 h prior to further processing. One parent specimen was initially prepared for each formulation. Test pieces were subsequently obtained from this specimen as required for irradiation, heat treatment, and characterization. These pieces originated from the same preparation. Representative photographs of the prepared samples are shown in Figure S1 of the Supplementary Materials.

### 2.3. Gamma Irradiation

The composites were irradiated in air at 25 °C using a ^60^Co gamma-ray source at a measured dose rate of approximately 3.0 kGy/h (3.03 kGy/h determined by PTW UNIDOS dosimetry) to a total absorbed dose of 100 kGy (irradiation time ~33 h). This dose was selected as a representative exposure level expected to induce measurable radiation-induced changes in the silicone-rubber composites. The extent and nature of these changes were evaluated using ATR-FTIR, EPR, and Shore A hardness measurements. This dose is comparable to levels employed in radiation-aging studies of silicone-based materials [7,8,20]. Unirradiated control samples were stored under identical environmental conditions and tested on the same schedule as the irradiated specimens. These controls served as references for the irradiation effects only; they were not subjected to the annealing treatment and therefore do not serve as controls for the annealing step.

### 2.4. Heat Treatment

The irradiated samples were heat-treated in a tube furnace (OTF-1200X; Hefei Kejing Materials Technology Co., Ltd., Hefei, China). Before heating, the furnace tube was purged with nitrogen for 10 min. A continuous nitrogen flow was maintained throughout the treatment to limit oxidation. Each sample was placed in the constant-temperature zone. Samples were heated at 10 °C/min to 60, 90, or 120 °C and held for 30, 60, 90, 120, 180, or 240 min. They were then cooled naturally to 25 °C under nitrogen.

The 60–120 °C range was selected to examine condition-dependent post-irradiation responses below the thermal-degradation onset reported for polydimethylsiloxane (PDMS) under inert atmospheres [21,22]. Published work also indicates that the post-irradiation chemistry of silicone-based materials can vary with temperature [6]. Holding times of 30–240 min were chosen to sample early, laboratory-scale evolution after irradiation. These temperatures and durations are experimental screening conditions and are not presented as service-equivalent exposures for marine nuclear components.

### 2.5. Characterization

#### 2.5.1. ATR-FTIR

ATR-FTIR measurements were performed nondestructively at 25 °C on one test piece per formulation and examined condition, derived from the corresponding parent specimen. ATR-FTIR spectra were recorded using a Nicolet iS50 spectrometer (Thermo Fisher Scientific, Madison, WI, USA) equipped with an ATR accessory. Spectra were collected from 4000 to 650 cm^−1^ at a resolution of 4.0 cm^−1^, with 32 instrumental scans averaged for each spectrum. These scans did not represent independent specimen replicates. A background spectrum was recorded before each measurement series. Transmittance spectra were converted to absorbance and then smoothed, baseline-corrected, and integrated using OMNIC software (version 9.8.286). Each band area was normalized to the total spectral area to account for variations in ATR contact and sample positioning. ATR-FTIR was used for qualitative band assignment and semi-quantitative comparison of normalized band areas. The normalized band areas of irradiated samples were compared with those of unirradiated controls; for annealed samples, the irradiated unannealed sample served as the reference. No absolute functional-group concentrations were derived.

#### 2.5.2. EPR

EPR measurements were performed only on SR-B_4_C because the high Pb content in the other composites attenuated microwave penetration and reduced resonator quality factor. For each examined condition, one EPR test piece was prepared from the corresponding material and measured once. For the irradiated and annealed states, preparation of the EPR test piece followed the respective treatment. Spectra were recorded at 25 °C on an EPR200-Plus spectrometer (CIQTEK, Hefei, China) at a microwave frequency of approximately 9.43 GHz, with a modulation frequency of 100 kHz and an amplitude of 2.00 G over a field range of 2000–4500 G. Each measurement comprised four instrumental scans averaged to obtain one spectrum; these scans did not constitute independent specimen replicates. After baseline correction and smoothing, the spectra were examined for resonance position, linewidth, and relative signal intensity. Sample masses and resonator Q factors were not used for normalization; signal intensities are therefore presented as relative values.

#### 2.5.3. Shore A Hardness

Shore A hardness was measured at 25 °C using an LXD-A durometer (Yueqing Hemu Instruments Co., Ltd., Wenzhou, China). The durometer method in GB/T 39693.4-2025 [23] (Rubber, vulcanized or thermoplastic—Determination of hardness—Part 4: Indentation hardness by durometer method (Shore hardness)) was used as the methodological reference. For each formulation and examined condition, three readings were obtained at different positions on one test piece derived from the corresponding parent specimen. The arithmetic mean and standard deviation of these three readings are reported as descriptive values. The three readings were repeated measurements on one test piece, rather than independent specimen replicates; the standard deviation represents variation among measurement positions.

#### 2.5.4. SEM and EDS

The surface morphology and elemental distribution of the unirradiated composites were examined using field-emission scanning electron microscopy (FE-SEM; Sigma 300, Carl Zeiss, Oberkochen, Germany) equipped with an energy-dispersive X-ray spectroscopy (EDS) detector (XFlash 760, Bruker, Dortmund, Germany). Prior to observation, the specimens were sputter-coated with gold. Backscattered electron (BSE) micrographs and corresponding EDS elemental maps were acquired at an accelerating voltage of 15 kV. The resulting images were analyzed qualitatively to assess the surface morphology and spatial distributions of Si, O, B, and Pb within the selected fields of view.

## 3. Results and Discussion

The three composites are designated as A (SR-B_4_C), B (SR-Pb-B_4_C), and C (SR-Pb). Unirradiated samples are denoted as A_0_, B_0_, and C_0_; irradiated samples without annealing as A_t__0_, B_t__0_, and C_t__0_; and annealed samples as A-t-m, B-t-m, or C-t-m, where t is 60, 90, or 120 °C and m is the holding time in minutes. The analysis focuses on changes within each formulation, as the formulations differ in silicone fraction, filler type, and total filler loading. Between-formulation differences are reported descriptively and are not interpreted as constituent-specific effects.

### 3.1. SEM Morphology and Elemental Distribution of Unirradiated Composites

**Figure 1:**
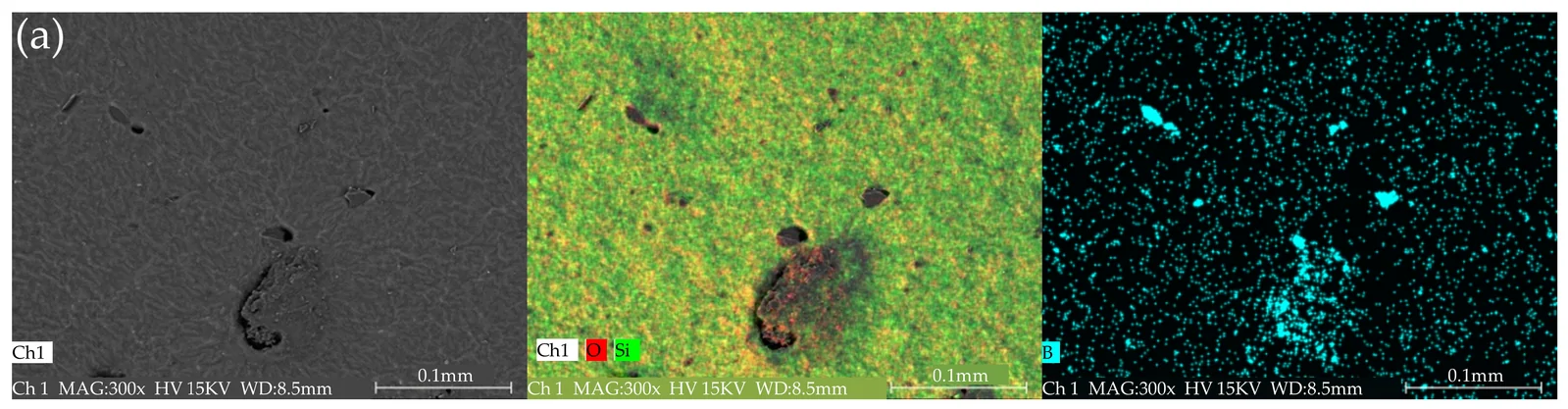

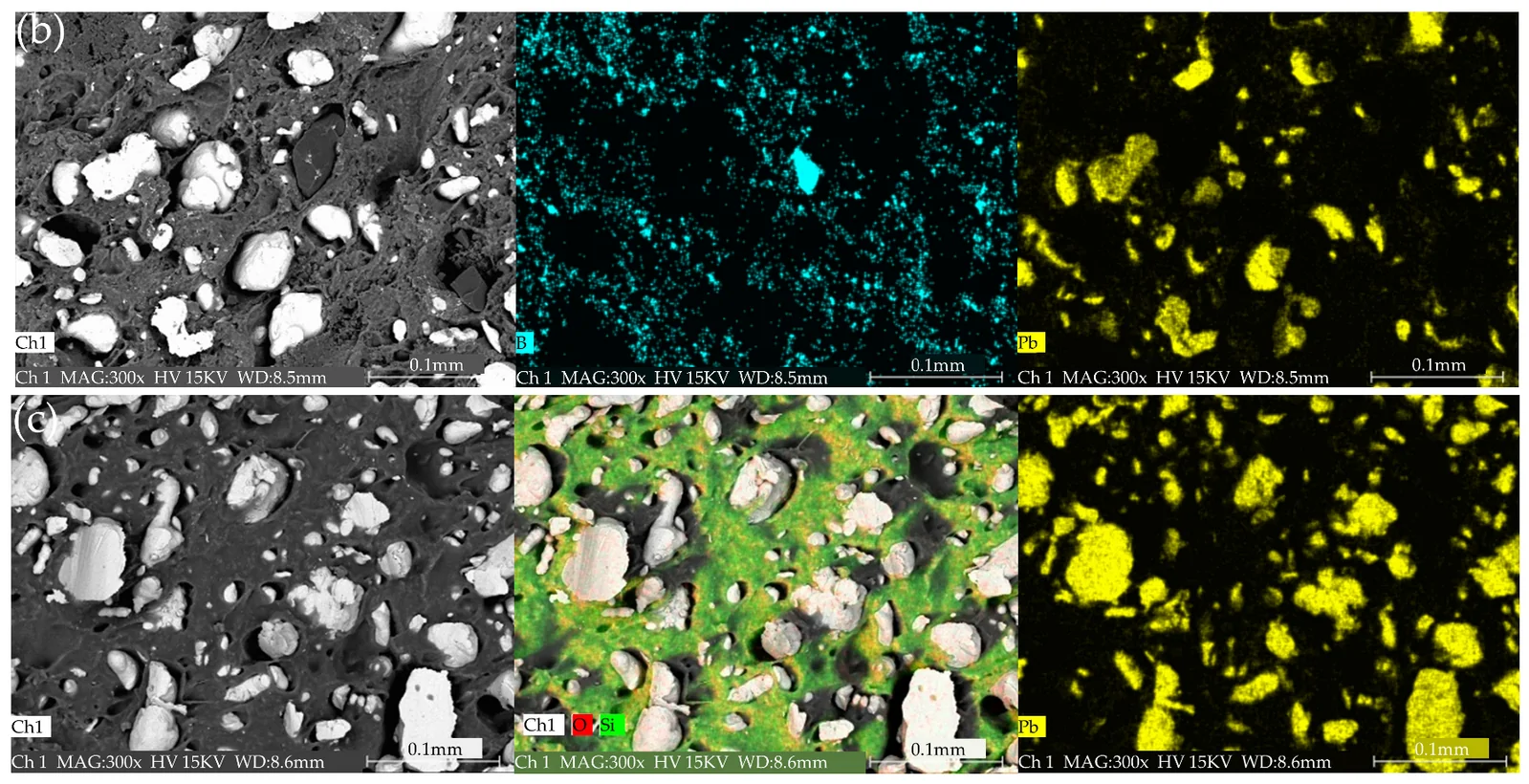
BSE micrographs and EDS elemental maps of the unirradiated composites. From left to right, (**a**) SR-B_4_C: BSE micrograph, Si/O elemental overlay, and B map; (**b**) SR-Pb-B_4_C: BSE micrograph, B map, and Pb map; and (**c**) SR-Pb: BSE micrograph, Si/O elemental overlay, and Pb map.

In unirradiated SR-B_4_C, the BSE image shows a relatively continuous matrix with several localized irregular regions (Figure 1a). The B map shows local enrichment consistent with B_4_C-rich regions, while the Si/O overlay extends across the surrounding phase. In SR-Pb-B_4_C, bright features in the BSE image coincide with concentrated Pb signals, supporting their identification as Pb-rich particles; the B signal is spatially uneven (Figure 1b and Figure S2). In SR-Pb, bright particle-like features likewise coincide with Pb-rich regions, while the Si/O overlay is observed in the surrounding phase (Figure 1c). These images describe selected fields of the unirradiated specimens and do not establish bulk filler dispersion or irradiation-induced morphological changes.

### 3.2. ATR-FTIR Analysis of Relative Spectral Response

ATR-FTIR spectroscopy was used as a qualitative and semi-quantitative method to examine each formulation in its unirradiated, irradiated, and post-irradiation-annealed states. Normalized band areas were obtained using the procedure described in Section 2.5.1. The resulting band-area ratios indicate relative changes in spectral distribution; they do not provide absolute concentrations of functional groups or chemical bonds. Peak overlap, surface heterogeneity, normalization, and ATR contact may also affect the reported ratios. Previous studies have used FTIR band intensities or areas to assess irradiation responses and silicone-rubber aging [24,25,26]. Normalization to the total spectral area and the same preprocessing procedure were applied to all spectra.

#### 3.2.1. Relative Spectral Responses After Gamma Irradiation

**Figure 2:**
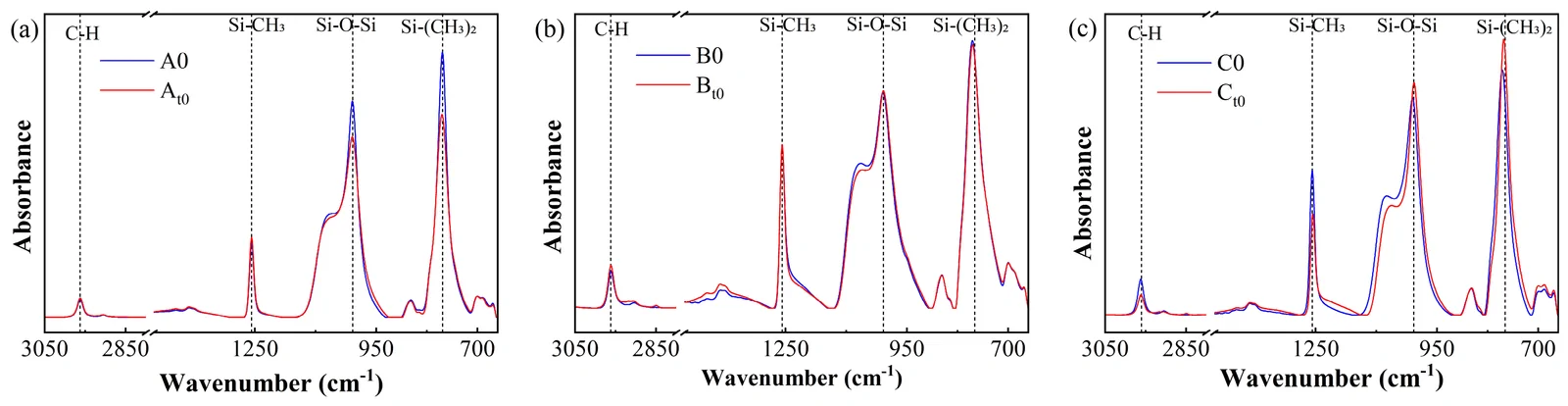
ATR-FTIR spectra of the composites before and after 100 kGy gamma irradiation: (**a**) SR-B_4_C; (**b**) SR-Pb-B_4_C; and (**c**) SR-Pb.

**Figure 3:**
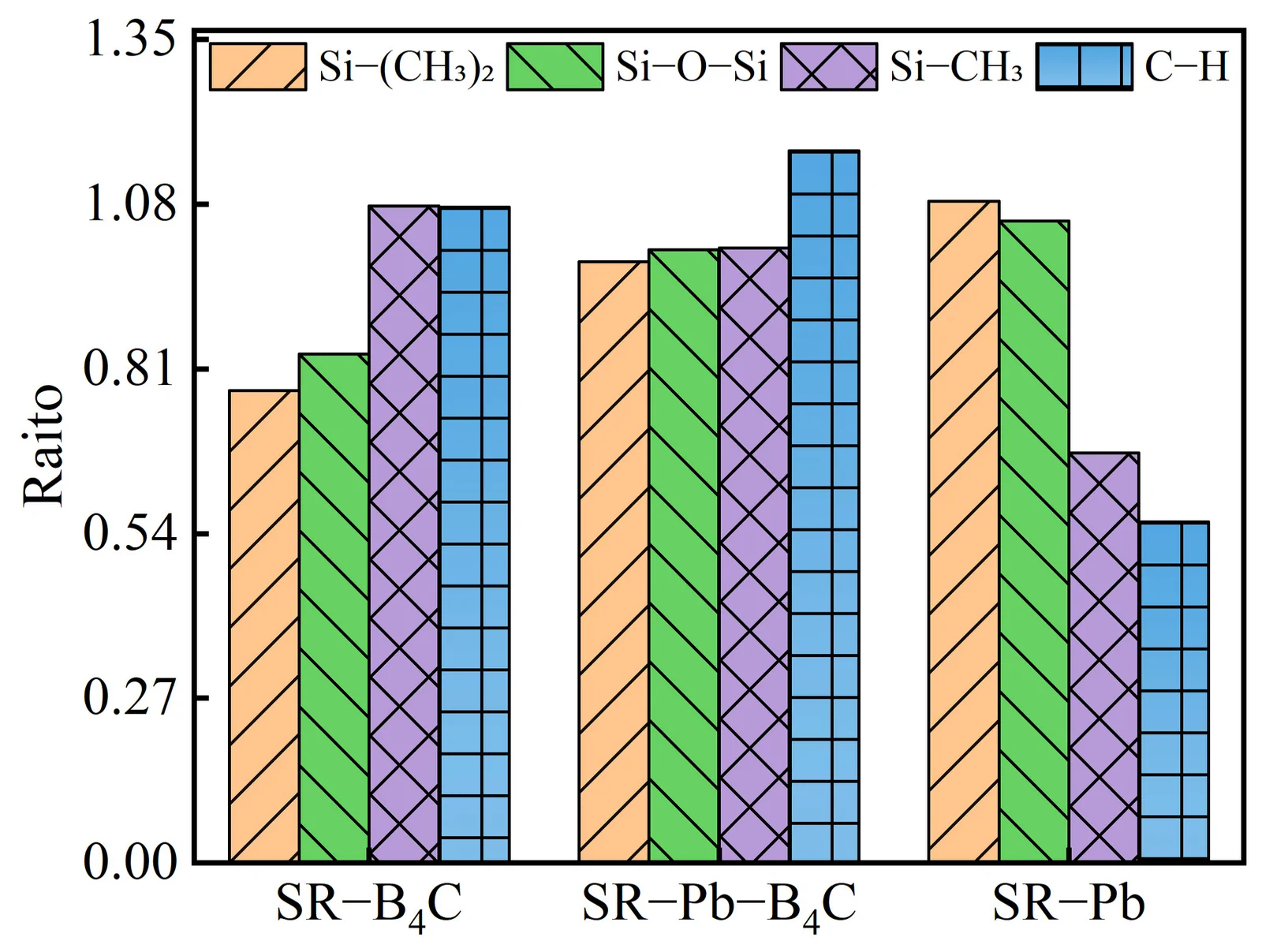
Ratios of the normalized ATR-FTIR band areas after 100 kGy irradiation to those of the corresponding unirradiated samples.

Figure 2 and Figure 3 present the ATR-FTIR spectra and normalized band-area ratios before and after gamma irradiation. All formulations retained the same characteristic infrared band positions after irradiation, although their normalized band areas changed relative to the corresponding unirradiated samples. These bands included C–H stretching at 2800–3050 cm^−1^, Si–CH_3_-related vibrations at 1240–1280 cm^−1^, Si–O–Si stretching at 1000–1130 cm^−1^, and Si–(CH_3_)_2_-related vibrations at 760–880 cm^−1^ [26,27]. No clearly resolved new band was observed after irradiation. The changes in normalized band areas document shifts in the relative spectral distribution and are reported as formulation-specific observations rather than evidence of a specific chemical mechanism.

For SR-B_4_C, the normalized Si–(CH_3_)_2_ and Si–O–Si ratios were below 1 after irradiation, whereas the Si–CH_3_ and C–H ratios were slightly above 1. For SR-Pb-B_4_C, the Si–(CH_3_)_2_, Si–O–Si, and Si–CH_3_ ratios remained close to 1, while the C–H ratio was above 1. For SR-Pb, the Si–(CH_3_)_2_ and Si–O–Si ratios were slightly above 1, whereas the Si–CH_3_ and C–H ratios were below 1. These changes are descriptive observations from one test piece per formulation and examined condition. The direction of change differed among the three formulations: SR-B_4_C showed a decrease in siloxane-related bands (Si–(CH_3_)_2_ and Si–O–Si), SR-Pb showed an increase in the same bands, and SR-Pb-B_4_C showed little change in most bands. Given that the ratios are close to unity and the measurements were obtained from a single specimen per condition, these small deviations may be influenced by variations in ATR contact, surface heterogeneity, and integration procedures.

At the same irradiation dose, the three formulations exhibited different within-formulation changes in normalized ATR-FTIR band areas relative to their respective unirradiated controls. The high-Pb formulations showed smaller deviations from unity in most band-area ratios than SR-B_4_C, but composition, silicone fraction, and total filler loading varied simultaneously. Literature on filled silicone systems provides several possible explanations, including restriction of matrix mobility by filler networks and normalization-related changes in band area [28,29,30,31]. The present data are compatible with radiation-induced modification of the silicone phase, but they do not identify chain scission, crosslinking, oxidation, or a filler-specific effect.

#### 3.2.2. Effects of Heat Treatment on the ATR-FTIR Bands

**Figure 4:**
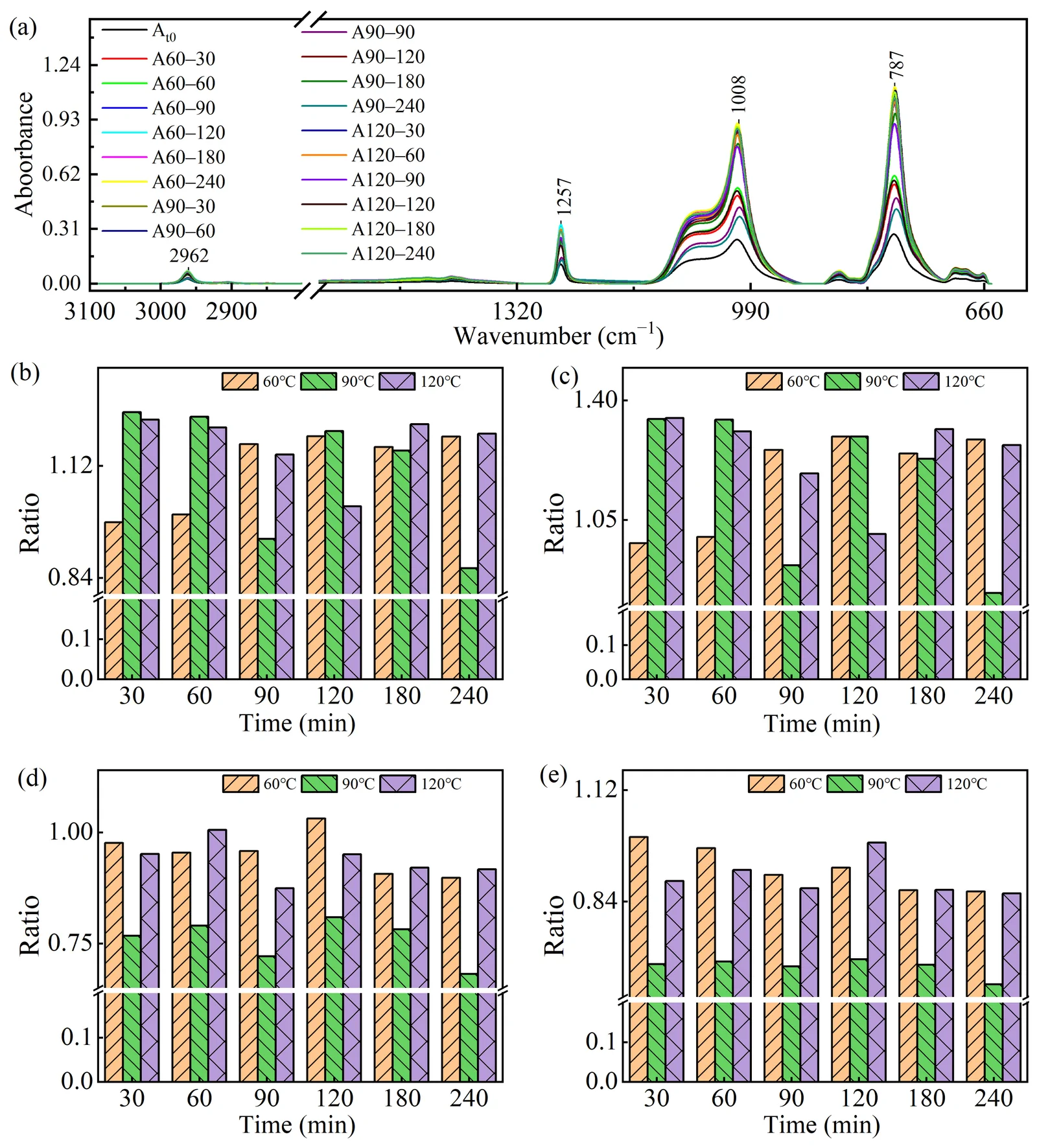
ATR–FTIR response of irradiated SR–B_4_C after heat treatment: (**a**) spectra; (**b**) Si–O–Si; (**c**) Si–(CH_3_)_2_; (**d**) Si–CH_3_; and (**e**) C–H normalized band-area ratios. Note: A_t0_ denotes the irradiated, unannealed sample. For the heat-treated samples, the notation At–m indicates the annealing temperature t (°C) and holding time m (min); for example, A60–30 represents annealing at 60 °C for 30 min.

**Figure 5:**
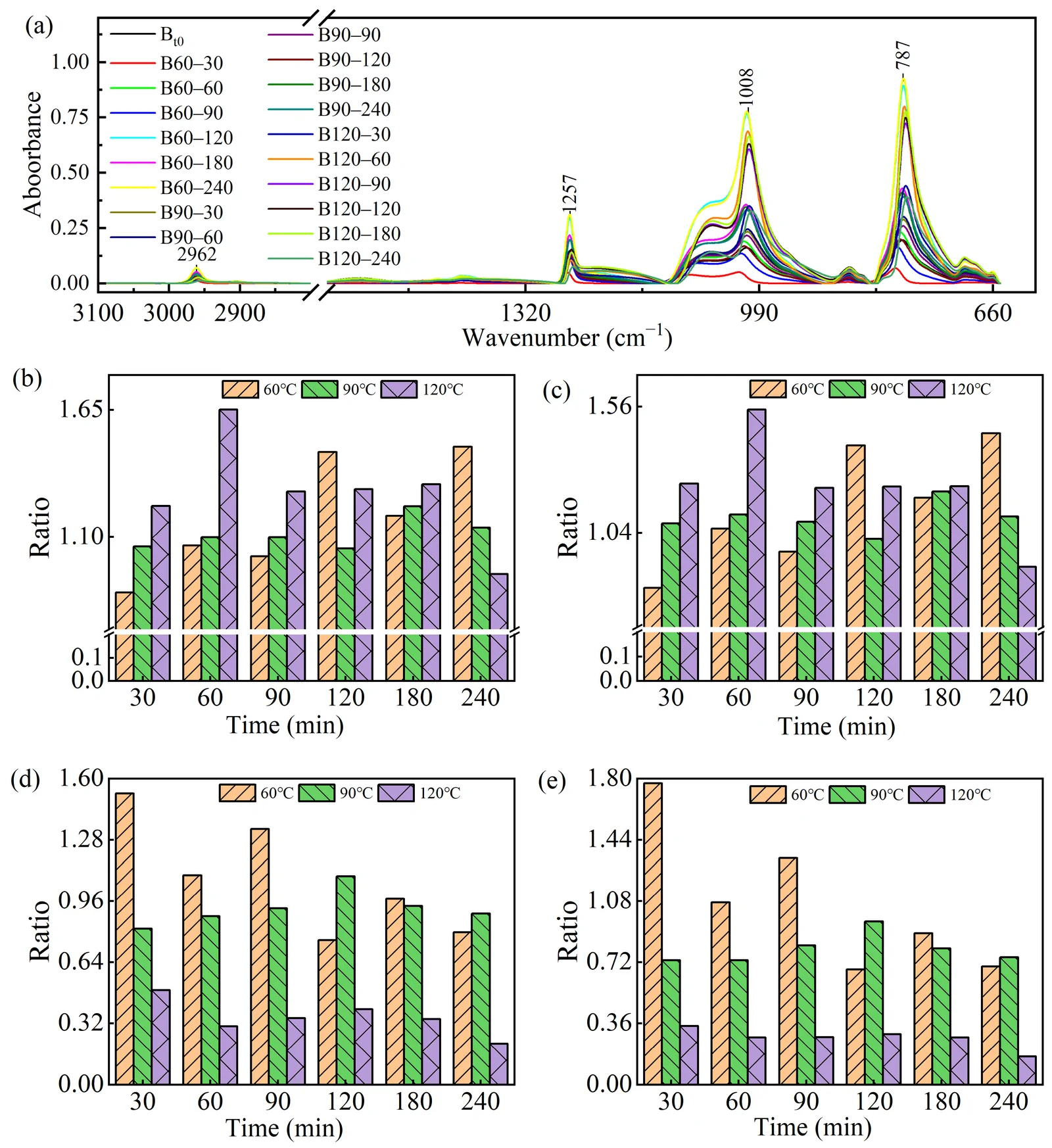
ATR–FTIR response of irradiated SR–Pb–B_4_C after heat treatment: (**a**) spectra; (**b**) Si–O–Si; (**c**) Si–(CH_3_)_2_; (**d**) Si–CH_3_; and (**e**) C–H normalized band-area ratios. Note: B_t0_ denotes the irradiated, unannealed sample. For the heat-treated samples, the notation Bt–m indicates the annealing temperature t (°C) and holding time m (min); for example, B60–30 represents annealing at 60 °C for 30 min.

**Figure 6:**
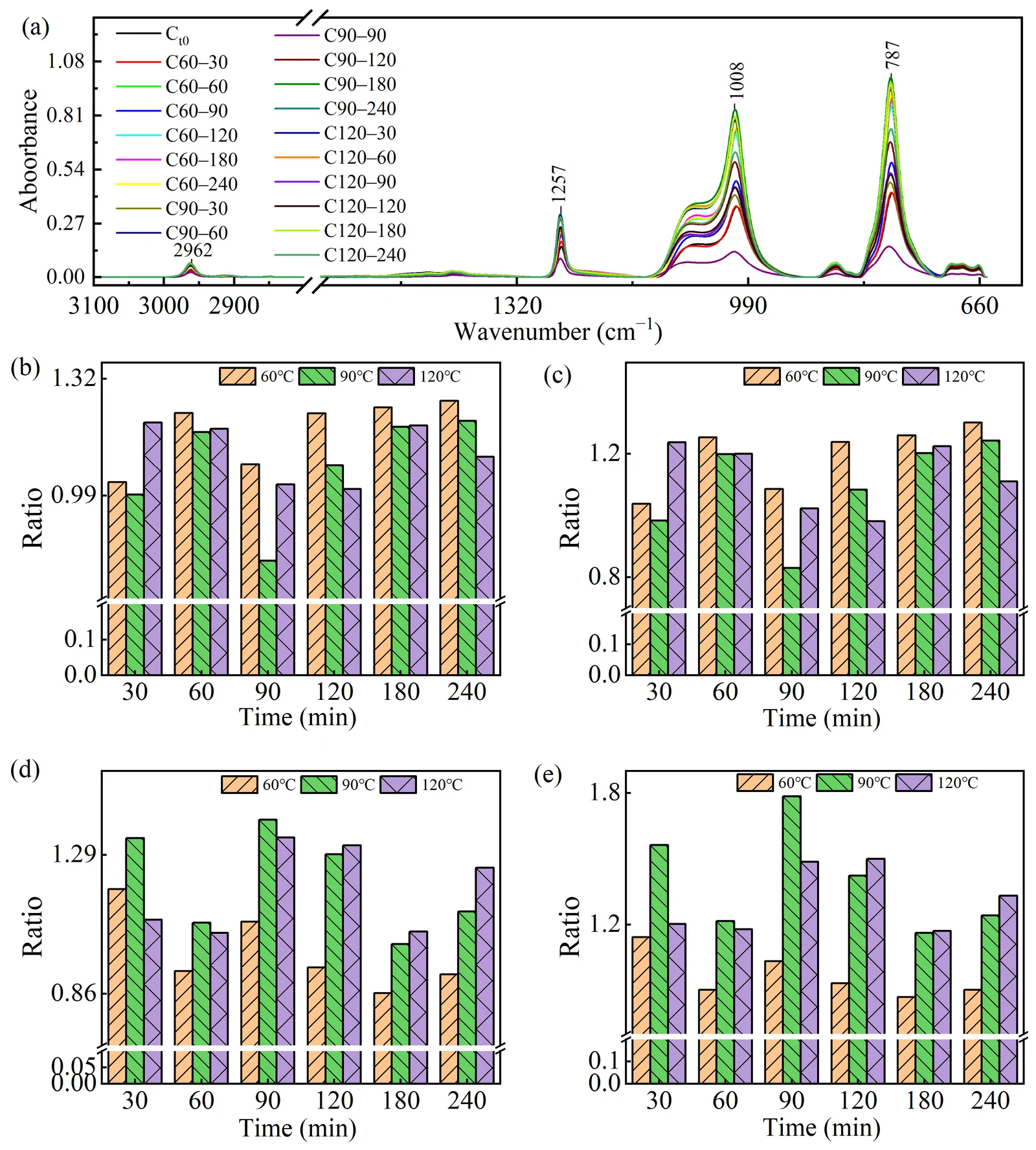
ATR–FTIR response of irradiated SR–Pb after heat treatment: (**a**) spectra; (**b**) Si–O–Si; (**c**) Si–(CH_3_)_2_; (**d**) Si–CH_3_; and (**e**) C–H normalized band-area ratios. Note: C_t0_ denotes the irradiated, unannealed sample. For the heat-treated samples, the notation Ct–m indicates the annealing temperature t (°C) and holding time m (min); for example, C60–30 represents annealing at 60 °C for 30 min.

The spectral changes induced by post-irradiation annealing were evaluated by comparing each heat-treated sample with its irradiated counterpart (without annealing), as shown in Figure 4, Figure 5 and Figure 6. Ratios above unity indicate an increase in normalized band area relative to the irradiated reference, while ratios below unity indicate a decrease. It should be noted that peak overlap, total-area normalization, and variations in ATR contact may influence these ratios; therefore, they are interpreted as relative rather than absolute changes.

For irradiated SR-B_4_C (Figure 4), several annealing conditions produced normalized Si–(CH_3_)_2_ and Si–O–Si ratios above the irradiated, unannealed reference, whereas most Si–CH_3_ and C–H ratios remained near or below unity. The increase in siloxane-related bands was more pronounced at 60 °C and 120 °C than at 90 °C (Figure 4b,c), suggesting a non-monotonic temperature dependence. Similar non-monotonic thermal responses have been reported for irradiated PDMS systems, where annealing can promote both crosslinking and chain rearrangement depending on temperature [6,30]. The side-group bands (Si–CH_3_ and C–H) did not show a corresponding increase under the same conditions (Figure 4d,e). This pattern is descriptively compatible with changes occurring primarily in the siloxane backbone rather than in the methyl side groups, but the present data do not establish this as a unique assignment. The absence of an unirradiated annealed control limits the ability to distinguish between annealing-induced relaxation of the irradiated network and additional reactions occurring during heating. The data are therefore reported as temperature- and time-dependent spectral variations within SR-B_4_C, rather than as evidence for a specific annealing mechanism.

For irradiated SR-Pb-B_4_C (Figure 5), several annealing conditions produced Si–(CH_3_)_2_ and Si–O–Si ratios above the irradiated, unannealed reference, whereas the Si–CH_3_ and C–H ratios were generally lower at 120 °C (Figure 5d,e). Thus, the siloxane-related bands and side-group bands showed opposite directional changes under the examined annealing conditions: the siloxane-related bands increased across multiple conditions, whereas the side-group bands decreased only at the highest temperature. The decrease in side-group responses at 120 °C is consistent with temperature-enhanced side-group reactions reported for silicone rubber [32], though the present data do not distinguish between crosslinking, degradation, or other pathways. The increase in siloxane-related bands was observed across a broader range of conditions than the decrease in side-group bands. The high filler loading may constrain the extent of network-level spectral changes, and the absence of an unirradiated annealed control limits the ability to resolve the thermal response from radiation-thermal coupling. The data are therefore reported as formulation-specific spectral responses to post-irradiation annealing, without inferring a specific reaction pathway.

For irradiated SR-Pb (Figure 6), most annealing conditions produced Si–(CH_3_)_2_ and Si–O–Si ratios above the irradiated, unannealed reference. Selected treatments at 90 and 120 °C also produced Si–CH_3_ and C–H ratios above 1 (Figure 6d,e). Thus, unlike SR-Pb-B_4_C, where side-group bands decreased at elevated temperatures, SR-Pb showed an increase in both siloxane-related and side-group bands under multiple annealing conditions. The increase in multiple band types is consistent with literature reports of annealing-induced structural rearrangement in silicone rubber [30,33], but the data do not distinguish among specific mechanisms. The absence of an unirradiated annealed control limits the ability to distinguish annealing-induced relaxation from other thermal processes. These observations are therefore reported as formulation-specific spectral responses to post-irradiation annealing, with the contrast between SR-Pb and SR-Pb-B_4_C noted descriptively rather than interpreted as a formulation-specific effect.

In summary, each formulation showed a distinct spectral response to post-irradiation annealing. SR-B_4_C showed increased Si–(CH_3_)_2_ and Si–O–Si ratios with little change in side-group bands. SR-Pb-B_4_C showed enhanced siloxane-related bands but decreased side-group responses at elevated temperatures. SR-Pb showed a broader response, with both siloxane-related and side-group bands increasing under multiple annealing conditions. These patterns are reported as formulation-specific observations rather than evidence for independent Pb or B_4_C effects.

### 3.3. EPR Analysis of Paramagnetic Centers in SR-B_4_C

**Figure 7:**
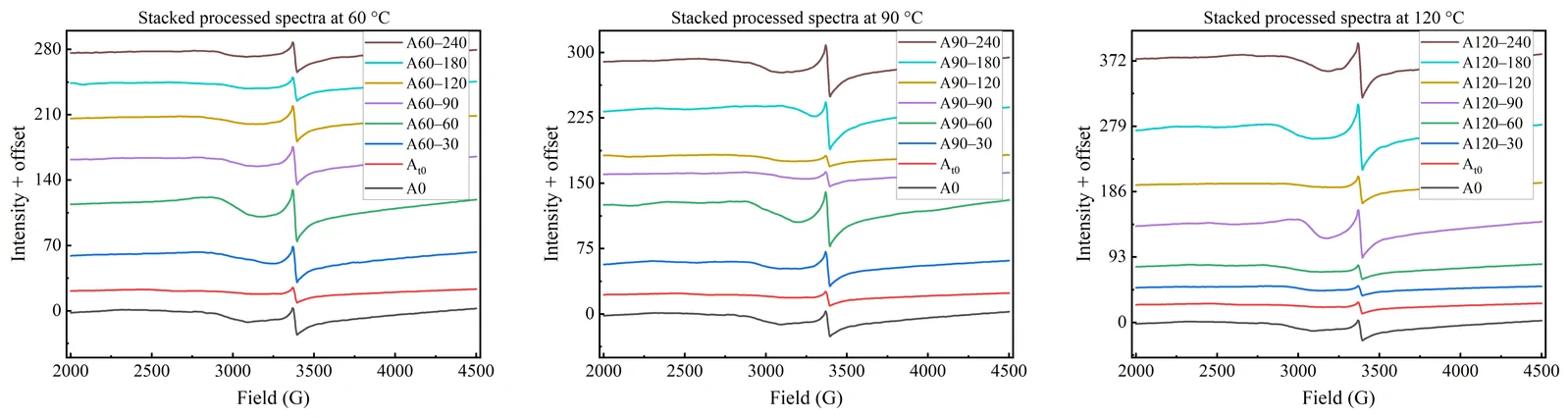
EPR spectra of SR-B_4_C before irradiation, after 100 kGy gamma irradiation, and after post-irradiation heat treatment.

Reliable EPR spectra were obtained only for SR-B_4_C because the high Pb loading in the other formulations attenuated microwave penetration and compromised resonator performance. Figure 7 presents SR-B_4_C spectra in the unirradiated, irradiated, and annealed states. All samples showed a main derivative signal between 3368 and 3398 G, without resolved hyperfine structure, with g-values of approximately 1.9935–1.9985 and linewidths of approximately 26.9 G. Signals near g = 2 have been reported for B_4_C defect centers [34,35]; however, the broad spectra do not establish that the dominant species remained unchanged across conditions.

A strong EPR signal was observed in the unirradiated SR-B_4_C sample, with a raw response exceeding that of the irradiated, unannealed sample. The measurements were not normalized to sample mass or resonator quality factor; signal intensities are therefore presented as relative values. The presence of a strong signal in the unirradiated state shows that the EPR response is not due solely to radiation-induced radicals in the silicone phase. B_4_C lattice defects and paramagnetic impurities are known sources of EPR signals [34], and irradiation of PDMS can generate several overlapping silicon-, oxygen-, and carbon-centered radical species [36]. The broad signal may thus contain contributions from B_4_C defect centers, interfacial species, and SR-derived radicals; the present spectra do not support assignment to a unique radical species.

The raw EPR response varied after heat treatment. At 60 °C, the signal increased during the first 30–60 min, decreased at intermediate times, and increased again at 240 min. At 90 °C, the signal was lower at 90–120 min relative to the initial value and increased again at 180–240 min. At 120 °C, the signal showed fluctuations, with local maxima at 90, 180, and 240 min. Because the spectra were not normalized to sample mass or resonator quality factor, these variations are presented as qualitative changes in the raw EPR response and cannot be interpreted as evidence of radical-decay kinetics or a causal relationship with hardness.

### 3.4. Effects of Gamma Irradiation and Heat Treatment on Shore A Hardness

**Figure 8:**
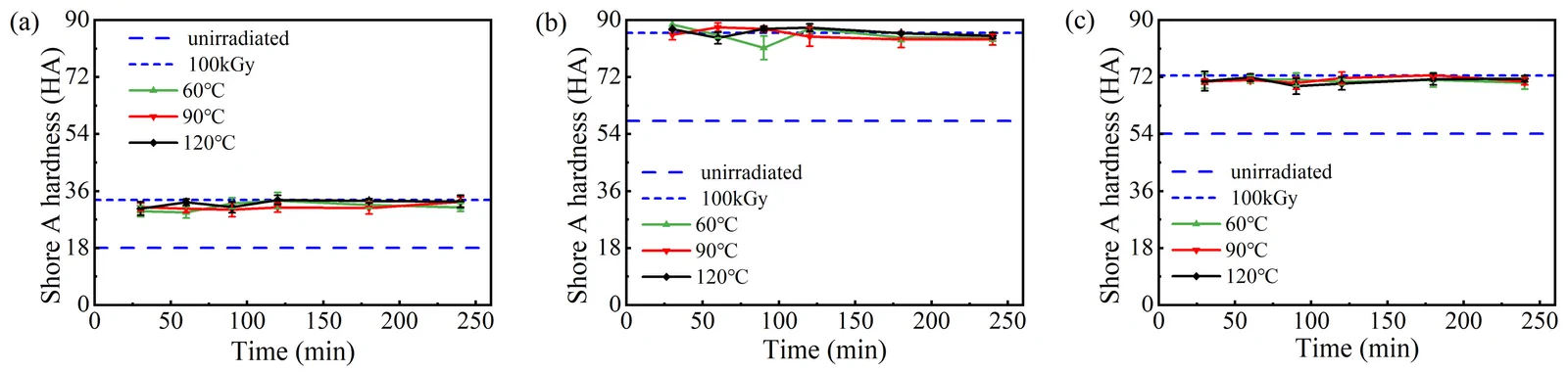
Shore A hardness of SR-B_4_C, SR-Pb-B_4_C, and SR-Pb before and after 100 kGy gamma irradiation and after post-irradiation heat treatment: (**a**) SR-B_4_C; (**b**) SR-Pb-B_4_C; and (**c**) SR-Pb.

Figure 8 presents the Shore A hardness of the three composites in the pristine state, after 100 kGy irradiation, and following post-irradiation annealing. Irradiation increased the hardness of all three formulations. The relative increases were about 83% for SR-B_4_C, 48% for SR-Pb-B_4_C, and 34% for SR-Pb. These increases indicate enhanced resistance to local indentation. However, hardness measurements alone do not by themselves identify changes in crosslink density or chain mobility. Irradiation-induced crosslinking and oxidation have also been linked to reduced elongation and increased rigidity in silicone-rubber products [20], but the present hardness data do not establish such mechanisms.

SR-B_4_C showed the lowest initial hardness, consistent with its lower total filler loading. Its mean hardness increased from approximately 18.1 before irradiation to 33.2 after irradiation. After annealing at 60–120 °C for up to 240 min, the values remained in the range of approximately 30–33, with only minor variation across the annealing conditions examined. Post-irradiation annealing therefore did not return the hardness to its pre-irradiation level.

The hardness of SR-Pb-B_4_C was substantially higher than that of SR-B_4_C. Its mean hardness increased from approximately 58.1 to 86.0 after irradiation. Annealing produced only a minor overall decrease, and the material maintained a high hardness. SR-Pb, containing 90 wt% Pb and 10 wt% SR, showed an initial hardness of approximately 54.1—lower than that of SR-Pb-B_4_C. Its mean hardness increased to approximately 72.5 after irradiation, and subsequent annealing induced only limited changes. In all three formulations, the hardness remained above the corresponding pre-irradiation level throughout the annealing conditions examined.

The three formulations differed in both absolute hardness and the magnitude of the irradiation-induced increase. SR-B_4_C showed the largest relative increase (83%), whereas the highly filled formulations (SR-Pb-B_4_C and SR-Pb) showed higher absolute hardness both before and after irradiation. The higher initial hardness of SR-Pb-B_4_C compared to SR-Pb was also observed. Factors that may contribute to these between-formulation differences include silicone fraction, filler type, total filler loading, particle dispersion, and interfacial adhesion, but these factors cannot be distinguished from the available data because they varied simultaneously. Overall, the main hardness change occurred after irradiation, whereas subsequent annealing produced comparatively small changes relative to the corresponding irradiated, unannealed samples. The hardness remained above the respective pre-irradiation values throughout the tested annealing window; this observation does not establish thermal irreversibility at the molecular level.

### 3.5. Possible Mechanisms of Irradiation-Induced Aging

SR composites may be subjected to both radiation and thermal exposure during service. Their aging behavior is governed by the competition between crosslinking and chain-scission reactions, which is further modulated by radical-mediated processes and the nature of the filler–matrix interface. Elucidating these interrelated processes is essential for predicting the service life of SR-based composites in radiation environments.

#### 3.5.1. Literature-Reported Radiation Pathways and Paramagnetic Signals

Gamma irradiation can ionize and excite SR molecules, leading to bond cleavage, radical formation, crosslinking, and chain scission [8,29,37]. In the present study, all three composites showed increased hardness after 100 kGy irradiation. ATR-FTIR spectra retained the characteristic SR band positions, although the normalized band areas varied with formulation. The broad EPR signal observed in SR-B_4_C was present even before irradiation and varied non-monotonically upon heating.

These observations are compatible with literature-reported pathways for irradiated silicone systems. Crosslinking and chain scission, for example, are known to compete during irradiation of SR, with crosslinking generally reported to dominate at lower doses or under inert atmospheres, whereas oxidative chain scission becomes more prevalent at higher doses or in the presence of oxygen [29,32]. Crosslinking typically increases hardness, while oxidative chain scission may lead to network softening [32]. Oxygen can also promote oxidation of methyl side groups, yielding volatile products such as formaldehyde and methanol [28,32], and irradiation may release small siloxane fragments, hydrogen, and methane [37,38]. However, crosslink density, gel fraction, volatile products, and oxidation products were not measured in this study. The observed hardness increase and spectral changes are therefore consistent with several possible pathways reported in the literature, but the present data do not distinguish which of these pathways, if any, were dominant in the investigated formulations. Moreover, the study did not include thermal analysis (e.g., thermogravimetric analysis (TGA), differential scanning calorimetry (DSC)), bulk mechanical testing (e.g., tensile/compression or dynamic mechanical analysis (DMA)), or shielding-attenuation measurements. The results therefore provide a description of the observed ATR-FTIR, qualitative EPR, and Shore A responses under the conditions examined, and do not establish thermal stability, recovery of flexibility, service life, or shielding effectiveness.

#### 3.5.2. Composition, Uncontrolled Variables, and Study Boundaries

The three formulations also differed in composition. SR-B_4_C contained 90 wt% SR and 10 wt% B_4_C; SR-Pb-B_4_C contained 20 wt% SR, 70 wt% Pb, and 10 wt% B_4_C; and SR-Pb contained 10 wt% SR and 90 wt% Pb. These compositional variations introduce additional factors that may affect the measured responses—such as Pb surface oxides, residual Pt from the Karstedt catalyst, metallic impurities in B_4_C, particle dispersion, and interfacial structure. The absence of unfilled silicone, compositionally matched formulations, unirradiated annealed controls, and independent specimen replication means that the present observations are formulation-specific; they are not interpreted as evidence for the independent effects of Pb, B_4_C, or their combination.

**Figure 9:**
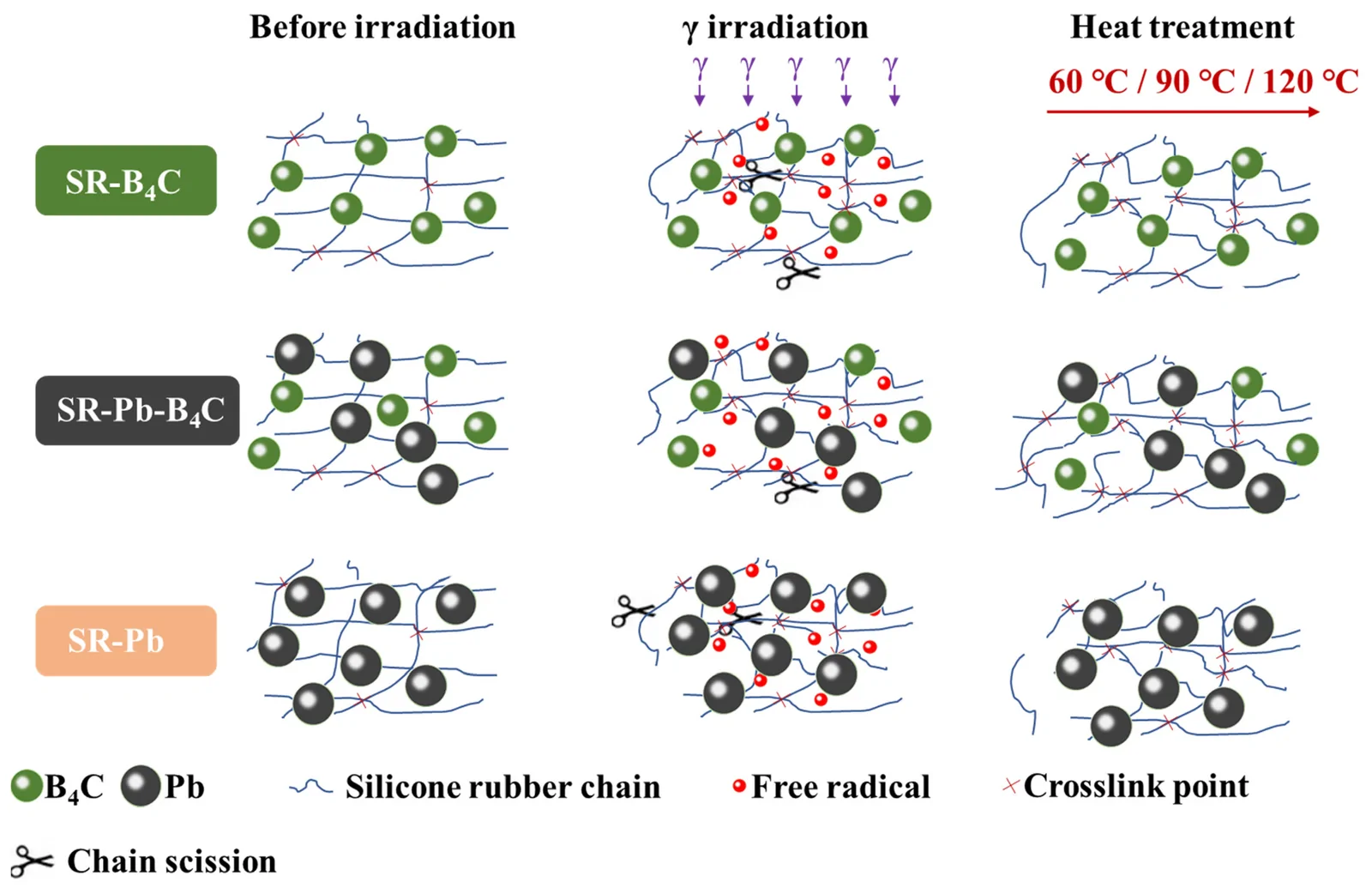
Proposed pathways for structural evolution of SR-B_4_C, SR-Pb-B_4_C, and SR-Pb during gamma irradiation and post-irradiation heat treatment. The scheme shows possible mechanisms and does not prove a unique reaction pathway.

Figure 9 summarizes selected literature pathways relevant to silicone-based composites under irradiation and post-irradiation annealing. Gamma rays are high-energy radiation that can cleave covalent bonds in the silicone phase, generating reactive sites such as radicals or ionic species. These reactive sites may subsequently undergo various radiation-chemical processes—including recombination, crosslinking, chain scission, or oxidation—depending on the local environment and the availability of oxygen. During post-irradiation annealing, the elevated temperature increases molecular mobility, which may accelerate reactions among the reactive sites. The presence of fillers such as B_4_C and Pb may further influence these processes by affecting radical migration, local energy deposition, or interfacial interactions. The scheme presents these pathways as literature-based possibilities and does not establish a mechanism demonstrated by the present data.

## 4. Conclusions

This study documented the responses of three predefined silicone-based composite formulations (SR-B_4_C, SR-Pb-B_4_C, and SR-Pb) to 100 kGy gamma irradiation and subsequent nitrogen-atmosphere annealing at 60–120 °C for 30–240 min. Within each formulation, the descriptive mean of within-sample Shore A hardness readings was higher after irradiation than before and remained above the corresponding pre-irradiation value throughout the annealing conditions examined. The relative increases were approximately 83% for SR-B_4_C, 48% for SR-Pb-B_4_C, and 34% for SR-Pb.

Post-irradiation annealing produced condition-dependent changes in the normalized ATR-FTIR band areas. The responses differed among the three formulations: SR-B_4_C showed increased siloxane-related bands (Si–(CH_3_)_2_ and Si–O–Si) with little change in side-group bands; SR-Pb-B_4_C showed enhanced siloxane-related bands but decreased side-group responses at elevated temperatures; and SR-Pb showed increases in both siloxane-related and side-group bands under multiple annealing conditions. The EPR spectra of SR-B_4_C showed qualitative variation with annealing temperature and time, with non-monotonic changes in signal intensity. These spectroscopic and indentation responses are reported as formulation-specific observations; they are not interpreted as evidence for specific radiation-chemical pathways or for independent Pb or B_4_C effects, because silicone fraction, filler type, and total filler loading all varied simultaneously among the three formulations.

The absence of recovery to pre-irradiation hardness values within the tested annealing window is noted as an empirical observation; it does not establish thermal irreversibility at the molecular level. The results provide a bounded description of the post-irradiation responses of the three composite formulations under the conditions examined.

Future studies should use compositionally matched formulations, appropriate irradiation and annealing controls, and independently prepared specimens to quantify changes in material properties and distinguish the effects of filler composition, loading, and treatment conditions. Comparisons between micrometer-sized and nanoscale fillers should be conducted at matched composition and loading, with particle dimensions and dispersion verified directly. Quantitative measurements of irradiation and annealing responses, mechanical properties, and photon and neutron attenuation would help assess whether trends observed in the present composites also apply to silicone-based shielding nanocomposites and identify any effects associated with filler size.

## Figures and Tables

**Table 1:** Mass composition and sample codes of the silicone rubber-based composites.

Formulation	SR (wt%)	Pb (wt%)	B_4_C (wt%)
A (SR-B_4_C)	90	0	10
B (SR-Pb-B_4_C)	20	70	10
C (SR-Pb)	10	90	0

Note: A, B, and C denote the sample group codes for SR-B_4_C, SR-Pb-B_4_C, and SR-Pb, respectively.

## Data Availability

The data that support the findings of this study are available from the corresponding author upon reasonable request.

## Supplementary Materials

The following supporting information can be downloaded at: https://www.mdpi.com/article/10.3390/nano16201286/s1, Figure S1: Prepared SR-B_4_C, SR-Pb-B_4_C, and SR-Pb composite samples; Figure S2: BSE micrographs and EDS elemental maps of 100 kGy gamma irradiation: SR-Pb-B_4_C.
