# Supplementary material for: Effects of Gamma Irradiation and Heat Treatment on the ATR-FTIR Response and Shore A Hardness of Silicone Rubber-Based Composites
Source: Nanomaterials (Basel). 2026 Oct 10;16(20):1286. doi: 10.3390/nano16201286 (PMC13707650; doi:10.3390/nano16201286)
Supplement: Supplementary file 1 [file nanomaterials-16-01286-s001.zip › nanomaterials-4561135-supplementary.pdf]

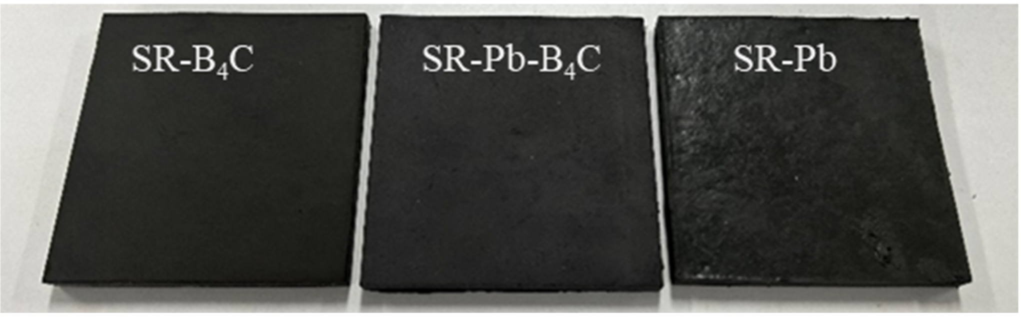

Figure S1. Prepared SR-B_4C, SR-Pb-B_4C, and SR-Pb composite samples.

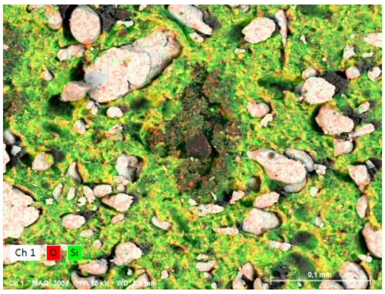

Figure S2 BSE micrographs and EDS elemental maps of 100 kGy gamma irradiation: SR-Pb-B_4C.
